# Variations in patient-reported physical health between cardiac and musculoskeletal diseases: systematic review and meta-analysis of population-based studies

**DOI:** 10.1186/s12955-015-0265-x

**Published:** 2015-05-30

**Authors:** James A. Prior, Kelvin P. Jordan, Umesh T. Kadam

**Affiliations:** Research Institute for Primary Care and Health Sciences, Keele University, Staffordshire, ST5 5BG UK; Health Services Research Unit, Keele University, Keele, UK

**Keywords:** Cardiovascular diseases, Chronic Disease, Musculoskeletal diseases, Physical health, Quality-of-Life

## Abstract

Population-based assessments of physical health are important to evaluate healthcare resource allocation. Normative data on the level of physical impairments attributable to specific diseases and severity levels within these diseases is critical to interpreting such data. Our objective, by means of a systematic review and meta-analysis, was to test the hypothesis that specific diseases which form cardiovascular and musculoskeletal disease spectra are associated with gradients of physical impairments. We examined a cardiovascular disease spectrum which consisted of hypertension, ischaemic heart disease and heart failure, and a musculoskeletal disease spectrum of lower back pain, osteoarthritis and rheumatoid arthritis. Using Medline, EMBASE and CINAHL databases, articles which had examined these morbidities and used either the SF-12 or SF-36 in general or primary care populations were selected; data was extracted independently by three reviewers. Study characteristics were described and the mean physical component summary scores of the SF-12 or SF-36 was analysed by disease, using random-effects meta-analysis. The association between disease and physical health (mean physical component summary scores) was assessed using multilevel meta-regression analysis, adjusting for age, health setting, country, disease definition and SF-12 or 36 format. From this search, 26 articles were identified, yielding 70 separate estimates of mean physical component summary scores across the morbidities from 14 different countries. For the selected conditions, pooled unadjusted mean physical component summary scores were: 44.4 for hypertension, 38.9 for ischaemic heart disease, 35.9 for heart failure, 39.5 for lower back pain, 36.0 for osteoarthritis and 36.5 for rheumatoid arthritis. The adjusted meta-regression showed mean physical component summary score difference for ischaemic heart disease of −4.6 (95 % confidence interval −6.0 to −3.2) and heart failure −7.5 (−9.1 to −5.9) compared to the hypertension category. For osteoarthritis −4.2 (−5.3 to −3.0) and rheumatoid arthritis −3.9 (−9.5 to 1.6) compared to the lower back pain category. Our findings provide the benchmark norms for the differences in physical health *within* and *between* disease spectra. Improved characterisation of the relative impact of individual conditions on physical health will facilitate public health assessments of chronic diseases as well as assessments of interventions using functional patient-reported outcomes.

**Electronic supplementary material:**

The online version of this article (doi:10.1186/s12955-015-0265-x) contains supplementary material, which is available to authorized users.

## Introduction

Research has shown the influence of specific chronic diseases on patients’ self-reported health status and found the magnitude of this to vary by disease [1, 2]. For example, both Ischaemic Heart Disease (IHD) and Osteoarthritis (OA) will adversely impact on physical health, but the relative impact of these two diseases in a patient population may vary [3, 4]. Chronic diseases often include conditions at differing stages of development, which means that each chronic disease is in fact a spectrum of conditions. IHD is only one example in a cardiovascular disease spectrum, ranging from hypertension to severe cardio or cerebrovascular conditions, which are linked by pathophysiology [5]. In contrast OA is just one example of a pain condition in a musculoskeletal disease spectrum, which can range from localised pain symptoms to more systemic conditions such as Rheumatoid Arthritis [6]. In this spectrum the links are based on the common symptom of pain, as opposed to shared pathophysiology.

For public health, the importance of this ‘spectrum’ approach is that it provides a population perspective of the range of conditions which may be associated with differing physical health impairments, with the implication that differing interventions may be required.

This conceptual approach of spectra is under-pinned by our previous clinical and epidemiological studies [7–9] and provides the opportunity to examine the concept of ‘relative severity’, i.e. how individual conditions influence health *within* the same chronic disease spectrum relative to one another [10]. This definition of ‘relative severity’ of different health conditions is based on previous GP focus groups, where the criteria for classifying severity were developed using clinical data and defined into four categories: i) chronicity and threat, ii) time course, iii) health care use, iv) impact on patients’ Activities of Daily Living (ADL)). Following this, a further 118 GPs took part in a consensus study to agree the severity definitions for a total of 188 morbidities. These methods were then tested in both English and Dutch general practice populations [8, 9].

Our rationale to examine different conditions, ranked *a priori* based on their severity within a spectrum is based on the knowledge that the ‘severity’ of a health condition frequently influences its clinical management. By understanding the state of a given condition within a spectrum, this can provide additional insight into a particular outcome and subsequent clinical decision-making. Therefore, knowledge of severity of conditions on a population level can add to the information aiding treatment and management choices.

Over recent decades, the measurement of overall health and physical health impairment using standardised questionnaires has become routine as a basis for measuring disease-related outcomes and focused on obtaining patient-centred outcomes data in international research and clinical practice. The most commonly used of such general health and well-being measures in population-based studies are the SF-12 & SF-36 health surveys [11]. The accumulation of such evidence provides the basis for benchmarking norms for the differences in physical health *within* and *between* chronic diseases. Synthesis of this evidence would provide population-based assessments of physical health for evaluation of healthcare resource allocation and the normative data that would be critical to interpreting such physical health data.

Whilst there is any number of spectra that could be tested under this hypothesis, the focus in this paper is on two spectra of common disease in populations, cardiovascular disease (CVD) and musculoskeletal disease (MSD) [6, 8]. To make the hypothesis testable and feasible, we *a priori* selected three specific morbidities for each spectrum and based their order of severity classification on our previous research. For the CVD spectrum; we chose the common and linked morbidities of hypertension, Ischaemic Heart Disease (IHD) and Heart Failure (HF) [12]. For the MSD spectrum, we chose the morbidities for which pain symptoms are a common experience, Lower Back Pain (LBP), Osteoarthritis (OA) and Rheumatoid Arthritis (RA) [13]. Using the three stated examples for the CVD and MSD spectra, we undertook a systematic review to examine the current evidence on the physical health status of these conditions, measured using the SF-12 or SF-36 [14, 15], in general populations or primary care populations. The objectives using systematic review and meta-analysis methods were; 1) to examine how physical health differs *within* a CVD and MSD spectrum and 2) to examine whether physical health differs *between* the two specified chronic disease spectrums.

## Methods

A systematic review, meta-analysis and meta-regression of identified research articles were conducted. Medical literature databases were searched to identify articles which included study samples defined with at least one of six specified conditions (hypertension, IHD and HF; and LBP, OA and RA), and which had reported a physical health outcome in the general population or primary care population. Selection of articles was based on standardised inclusion and exclusion criteria. Initial comparisons of the mean PCS scores were made using meta-analysis; a meta-regression was used to examine pooled and adjusted estimates for each condition and compared *within* and *between* the two spectrums.

### Chronic disease spectra

For CVD, the systematic review focused on hypertension, IHD and HF articles. Where an article included patients who had been defined as having IHD, angina or myocardial infarction (MI), all were defined within the IHD category. For MSD, the focus was on LBP, OA and RA articles. Within each spectra, use of the three conditions is justified for such examination based on their distinct severity and symptom profiles at a population level. Conditions were placed in *a priori* order of severity, based on previous clinical and validation studies in English and Dutch populations [7, 8] and the distinct spread of symptom experience for these conditions within their respective chronic diseases meant that conditions could be ordered from ‘less severe’ (i.e. hypertension for CVD and LBP for MSD) to those defined as ‘moderately severe’ (i.e. IHD for CVD and OA for MSD), and to ‘most severe’ (i.e. HF for CVD and RA for MSD).

### Article selection

Standard search strategies were carried out in the three databases of Medline, EMBASE and CINAHL, using the National Health Service (NHS) evidence webpage (https://www.evidence.nhs.uk/). Selection of an article into the final review was based on inclusion criteria which included; (i) study samples being aged ≥18 years, (ii) recruited either from the general population or primary care and (iii) defined as having one of the six study specified conditions of interest. Only observational studies were included in the review.

The primary measure of physical health for studies in this review was the Physical Component Summary (PCS) score from either the SF-12 or SF-36. Both the SF-12 & SF-36 surveys form aggregate scores of general physical health, the PCS score and general mental health, the Mental Component Summary (MCS) score. These scores are normalised to a general US population mean of 50 (Standard Deviation (SD) 10), scores below 50 indicate worse physical or mental health than the ‘average’ US population [16]. The use of these summary scores across the selected articles allowed the comparison of different conditions in populations [17–20].

Exclusion criteria were (i) specifically sampling patients only aged 17 years or under, (ii) articles published pre-1990 (these SF surveys were developed from the late 1980s and not used prior to the 1990s [14]), (iii) related to a randomised control trial (RCT) design, or iv) non-English language articles due to limited resources for translation. In articles including both baseline and follow-up longitudinal data from observational studies, only the baseline data was included into the analysis.

### Search and selection strategies

This systematic review implemented nine search strategies in total, as a result of conducting three chronic disease specific searches (“cardiovascular”, “musculoskeletal” and “chronic”) in the three literature databases (Additional file 1: Table S1a-c). Each search strategy was based on ‘exploded’ Medical Subject Heading (MeSH) terms and individual keywords.

The selection of articles was based on; title screening, abstract screening, and then review of the full article, using a total of three reviewers (JAP, KPJ, UTK). Titles were screened by the first reviewer (JAP), and the list of abstracts for inclusion or exclusion generated. Two reviewers (UTK & KPJ), blinded to the abstract selections of the first reviewer, completed the abstract selection by reviewing half the total number of abstracts each. Disagreements on inclusion or exclusion of articles were resolved using a triangulation process.

The full articles included in the review underwent structured data extraction. Each article could have examined the influence of several of the six specified conditions of interest. The mean PCS scores for each condition, and a measure of distribution around the mean; (Standard Deviation (SD)), were extracted. Other information extracted included: the lead author and publication year; sample size, along with their mean age (SD), the health care setting (general population or primary care), method of condition definition, samples’ country and SF format used (SF-12 or SF-36). In the instances where an article was included in the review, but the required data were not reported, the corresponding author was contacted and the data requested directly.

### Data synthesis & analysis

Mean PCS scores for each condition were initially compared through meta-analysis and presented as forest plots. For each condition, articles were ordered by the region from which they came; Europe (subdivided into Northern, Eastern, Southern or Western Europe), North America or Asia. These forest plots provided a graphical representation of the mean PCS score by; i) article, ii) condition and iii) each chronic disease overall. This unadjusted analysis used a random-effects meta-analysis model. For IHD, where separate estimates for ‘IHD only’, ‘angina’ and ‘MI’ were given in a study, these were combined where appropriate.

Multilevel meta-regression [21] was used to compare the pooled mean PCS scores between the conditions. This method takes into account the potential clustering effects within studies examining more than one of the selected conditions (for example, patients in both the hypertension and heart failure condition categories).

A separate meta-regression was conducted for CVD and MSD to examine the *within* spectrum association with mean PCS score (reference categories were; hypertension for CVD & LBP for MSD). Following that, a further meta-regression was conducted to examine the *between* spectra association with mean PCS score compared to the reference category (hypertension) mean PCS score. Difference in PCS scores from the reference category in each set of analysis were defined as a ‘Minimal Clinically Importance Difference’ (MCID) if the PCS score was 3 points or greater [22]. Meta-regressions were adjusted for age, health setting, country, method for defining condition and format of the SF survey. Each of these variables represents a potential source of variation in the SF estimate.

Each variable was dichotomised, these binary variables included; mean dataset age (≤59 or ≥60 years); health setting (general population or primary care); country (Europe or ‘Rest of the world); method of condition definition (self-report or ‘other method) and finally, whether the SF-12 or SF-36 had been used. *Within* spectrum meta-regression was reported as i) unadjusted values, ii) adjusted for age and iii) adjusted for all other stated variables mentioned above. *Between* spectra meta-regression is only reported with adjustment for all stated variables. Analyses were performed using STATA/IC 12.1 for Windows and MLwiN v.2.22 [23].

## Results

### Article selection

**Fig. 1:**
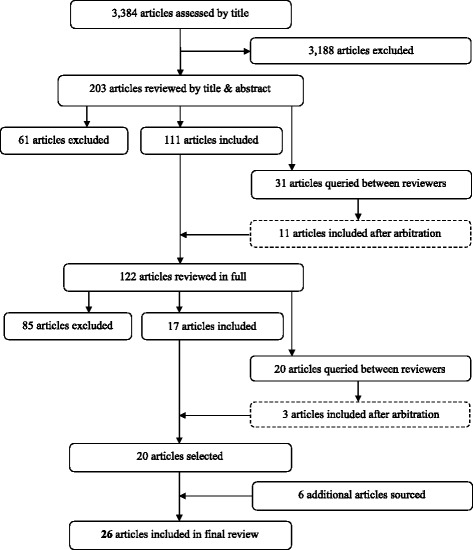
Selection of studies for inclusion in review

From the total of 3,384 unique articles identified, after exclusion, 122 were reviewed in full. From these 122 articles, 20 met the inclusion criteria and an additional six articles were identified through the reference lists of the included 20. This process resulted in 26 articles finally being included in the systematic review (Fig. 1).

The 26 articles included in this review were from 14 different countries, incorporating a total of 70 estimates of mean PCS scores across the six specified conditions and for a total population of 43,840 individuals. For the CVD spectrum, this review identified 22 estimates for hypertension [4, 10, 24–36], 19 estimates for IHD [4, 10, 27, 33, 36, 37] and 12 for HF [10, 27, 36, 38, 39]. CVD studies were predominantly from European countries, with several also from the US & Asia. For the MSD spectrum, six mean PCS estimates for LBP [4, 10, 31, 40–42], seven for OA [3, 10, 31, 33, 41, 43] and four for RA [25, 44–46] were identified. MSD studies were predominantly from European countries, with several also from the US.

At the point of meta-analysis, IHD articles having examined more than one IHD sample  (i.e. MI & angina) were pooled. Therefore, a total of 64 estimates of mean PCS scores were finally used in the meta-analysis and meta-regression, 13 of which were categories as IHD, condensed from the original 19.

### CVD spectrum and physical health

The 22 estimates of PCS scores in hypertension samples came from 15 articles in 12 countries, with a combined size of 27579. The mean ages of these samples ranged from 42.9–68.1 years, with just over half from the general population. The 19 estimates of PCS scores in IHD samples came from six articles in 11 countries with a combined size of 3641. Half of these came from the general population with mean ages ranging from 43.3–70.2 years. For HF there were 12 estimates of PCS scores from five articles in eight countries with a combined sample of 1985. Mean ages of these samples ranged from 57.4–76.5 years, with two thirds from general population samples (Additional file 2: Tables S2a-c).

**Fig. 2:**
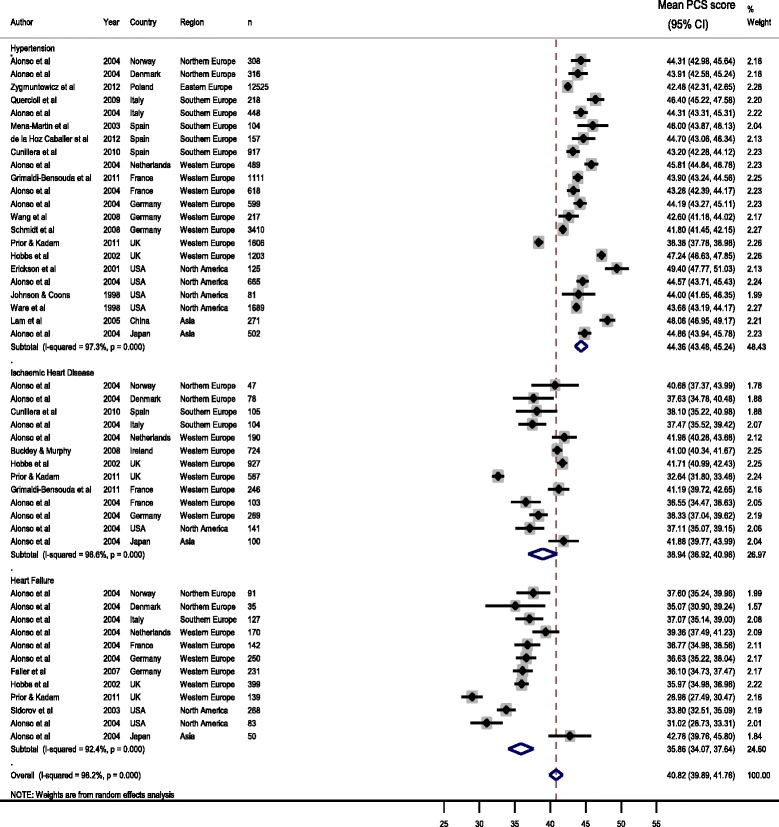
CVD forest plot

Estimates from the initial meta-analyses showed the following summary scores: for hypertension pooled mean PCS score was 44.4 (95 % CI 43.4 to 45.2); IHD was 38.9 (36.9 to 41.0) and for HF was 35.9 (34.1 to 37.6) (Fig. 2).

### Meta-regression of CVD studies

The unadjusted meta-regression showed an association between increasing CVD severity and poorer physical health (Table 1). Compared to the US general population mean of 50, the unadjusted mean PCS score was six points lower for hypertension, 11 points lower for IHD, and 14 points lower for HF. When adjusted for age, differences between the CVD conditions remained, but did decrease in magnitude. With hypertension as the reference group, the age-adjusted pooled mean PCS score for both IHD (−4.6 (−5.9 to −3.2)) and for HF (−7.3 (−9.1 to −5.9)) were significantly lower. The final stage of adjustment for health setting, country, condition definition and SF-12 or 36 formats had minimal influence on these associations, which remained significant.

**Table 1:** Comparison of pooled mean PCS scores between conditions

		*Within* spectrum analysis^a^			*Between* spectrum analysis^a^
Condition category	Unadjusted Mean PCS score (95 % CI)	Unadjusted difference in mean PCS score (95 % CI)	Age adjusted difference in mean PCS score (95 % CI)	Adjusted^b^ difference in mean PCS score (95 % CI)	Adjusted^b^ difference in mean PCS score (95 % CI)
**Cardiovascular diseases**					
Hypertension	44.4 (43.5 to 45.2)	Ref	Ref	Ref	Ref
Ischaemic Heart Disease	38.9 (36.9 to 41.0)	−5.3 (−6.4 to −4.1)	−4.6 (−5.9 to −3.2)	−4.6 (−6.0 to −3.2)	−4.8 (−6.2 to −3.5)
Heart Failure	35.9 (34.1 to 37.6)	−8.2 (−9.5 to −7.0)	−7.3 (−8.9 to −5.7)	−7.5 (−9.1 to −5.9)	−7.4 (−8.9 to −5.9)
**Musculoskeletal diseases**					
Lower Back Pain	39.4 (35.9 to 43.0)	Ref	Ref	Ref	−1.7 (−3.6 to 0.2)
Osteoarthritis	36.0 (33.3 to 38.6)	−4.3 (−5.4 to −3.2)	−4.2 (−5.3 to −3.1)	−4.2 (−5.3 to −3.0)	−5.1 (−6.9 to −3.3)
Rheumatoid Arthritis	36.5 (33.6 to 39.4)	−2.7 (−4.3 to −1.1)	−4.1 (−7.9 to −0.3)	−3.9 (−9.5 to 1.6)	−6.0 (−9.5 to −2.5)

^a^accounting for clustering within studies using multilevel meta-regression

^b^Adjusted for: age, health setting, location, method of disease definition & SF-12 or SF-36 format

### MSD spectrum and physical health

The six estimates of mean PCS scores for LBP were from separate articles from six different countries, with a combined size of 2433. Mean ages ranged from 49.9–81 years, with four drawn from primary care. There were seven estimates of mean PCS scores for OA from seven articles, in six different countries with a combined size of 7582. Mean ages ranged from 43.3–71.0 years, with all but one study conducted in primary care. Finally, the four estimates of mean PCS scores for RA came from four articles in three countries with a combined size of 2620. Three of the four were based in primary care and mean ages ranged from 53.9–61.3 years old (Additional file 2: Tables S2d-f).

**Fig. 3:**
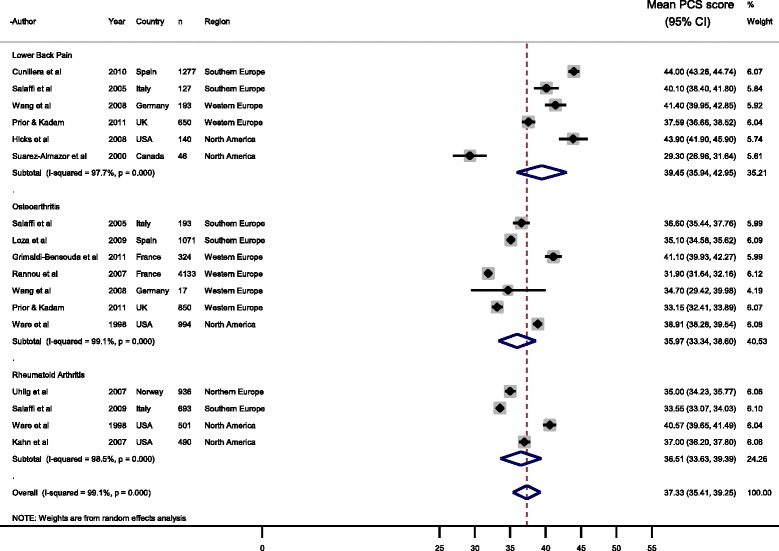
MSD forest plot

Estimates from the initial meta-analyses showed the following summary scores: for LBP pooled mean PCS score was 39.4 (35.9 to 43.0); OA was 36.0 (33.3 to 38.6) and for RA was 36.5 (33.6 to 39.4) (Fig. 3).

### Meta-regression of MSD studies

The unadjusted meta-regression showed similar associations of OA and RA having poorer physical health compared to the LBP category. Compared to the US average, the unadjusted mean PCS score was 10 points lower for LBP, 14 points lower for OA, and 13 points lower for RA. With LBP as the reference group, the age-adjusted pooled mean PCS score for both OA (−4.2 (−5.3 to −3.1)) and RA (−4.1 (−7.9 to −0.3)) was significantly lower. Further adjustment for health setting, country, condition definition and SF-12 or SF-36 format had minimal influence on the magnitude of associations with OA, but the association for RA became non-significant (Table 1).

### Comparison between spectrums

Compared to hypertension, the adjusted pooled mean PCS score for LBP was non-significantly lower by −1.7 (−3.6 to 0.2), whilst OA was significantly lower by −5.1 (−6.9 to −3.3) and RA significantly lower by −6.0 (−9.5 to −2.5). The adjusted estimates of pooled PCS scores for hypertension and LBP were similar, as were those of IHD and OA, and for HF and RA (Table 1).

## Discussion

### Overall findings

This systematic review and meta-analysis provides population-based estimates of physical health limitations associated with cardiovascular and musculoskeletal diseases from international studies. For CVD, the gradient in associations from hypertension to IHD to HF, provide an indicative health status of populations and the potential change in physical health across the CVD spectrum as populations go from a single cardiovascular disease (such as hypertension) to more complex end-stage conditions such as heart failure. For the MSD spectrum, there was less of a gradient in the three chosen conditions which indicates that associated poor health might not develop across the pain-spectrum conditions as hypothesised.

Within the CVD spectrum, the mean PCS score estimates of hypertension and IHD were comparable to those of the same disease groups from US normative disease group data. Our estimates for hypertension 44.4 and for IHD 38.9, were comparable to those reported in the derived SF-12 US normative median values of 45.5 and 38.1 respectively [47]. Such similarity of these crude PCS scores suggests our estimates provide an accurate baseline from which to consider the adjusted differences between conditions.

After adjustment for age and other study-design factors, the differences within the selected CVD spectrum were greater than 3 points, which can be considered a minimal clinically important difference [22, 48]. Such differences provide the public health estimation of physical health impairments within the CVD spectrum as these related conditions develop. In current public health approaches, interventions are tailored to the CVD spectrum for the reduction in mortality outcomes [49, 50]. Therefore, such an existing framework could be applied to the characterisation of relative impact of chronic diseases on physical health. This will facilitate public health assessments of chronic disease progression as well as assessments of interventions using functional patient-reported outcomes. For example, population level benchmark norms could be used to produce clinical guidance on when interventions to improve physical health should be introduced during the course of a progressing chronic disease, such as CVD.

Whilst all three selected MSD were associated with poorer physical health compared to the general US population, the relative differences between the conditions were less distinct than the CVD spectrum. It is arguable here that public health approaches to these types of MSD need to focus on the pathology of the pain condition as opposed to quality of life in such populations [51–53]. Though the relative adjusted difference was deemed clinically important and significant for OA and RA (4 points lower) compared to LBP, crude estimates were not comparable to US disease specific norms. Our crude estimate for LBP was much poorer (10 points) than mean scores determined for the same disease group from normative US data (39.4 *vs*. 48.6 respectively). Estimates for OA & RA were also poorer than normative disease specific data. However, we did observe a similar impact on mean PCS score estimates for OA & RA (36.0 & 36.5 respectively) which was also shown by the US normative disease specific data (39.0 & 40.9 respectively) [47]. Here the findings suggest a severe, but similar influence of the MSD conditions on physical health, but do not support the MSD gradient.

In public health terms, the importance of these findings here is that for this CVD spectrum we have quantified a gradient of physical health in different conditions. Though it could be argued that comorbidity is contributing to such poor physical health, it still remains that populations with serious CVD such as heart failure will have poorer health, no matter the direct cause. However, for MSD, it may be the experience of any MSD condition that is important and not necessarily what type.

The findings *between* chronic disease spectra suggest that the associations with poor physical health status can be similar in populations that might experience CVD or MSD. The adjusted estimate of physical health score for LBP was not statistically different from hypertension, IHD was equivalent to OA, and HF was similar to RA. Such results give additional support to our constructed spectra, as results are comparable across the ‘less’, ‘moderately’ and ‘most’ severe conditions displaying similar impacts on physical health. In public health terms, the importance of these comparable findings is the suggestion that, on a population level, any CVD and MSD conditions of a similar ‘severity level’ may be important in the influence on physical health and not necessarily the specific type of condition.

### Strengths and limitations

This systematic review and meta-analysis identified the key international studies that had investigated the association between chronic disease and physical health measured using a standardised instrument. This was achieved by the *a priori* test of relative severity, ordering diseases within the same chronic disease spectrum. The selection of the two spectra, both formed from common conditions, provides the comparison of physical health estimates and the practical approach to devising comparisons *within* and *between* different chronic diseases.

In using data drawn from international studies, the caveat is that heterogeneity for the selected condition categories might be the partial explanation for some of the differences identified, despite the adjustment for the study design and setting. Whilst, specific conditions such as heart failure, ischaemic heart disease, osteoarthritis and rheumatoid arthritis have defined diagnostic criteria [54–57], conditions such as hypertension and lower back pain criteria might differ. The scope of the review was international and most studies were of an appropriately large sample size, however the exclusion of articles not written in English, might have introduced selection issues. It could be argued that the MSD examples had reduced power to detect the range of physical impairments because of the small number of studies identified. Finally, though each condition in the CVD or the MSD spectrum were indicators of the likely physical impairment, the analyses did not account for comorbidity, other than adjusting by age as a proxy marker. Conditions such as heart failure or rheumatoid arthritis often accrue comorbidity as a result of age and complication, and the physical impairment therefore may in part be a reflection of the index HF or RA, or also any associated comorbidity.

## Conclusions

Our systematic review and meta-analysis describes the relative physical limitations associated with spectra of cardiovascular and musculoskeletal diseases. The findings provide benchmark norms for the differences in physical health both *within* and *between* diseases. Improved characterisation of the relative impact of health conditions on physical health will facilitate public health assessments of chronic disease, as well as population-based assessments of clinical interventions using functional outcomes which are meaningful to patients.

## Additional files

Additional file 1: Table S1a. Cardiovascular disease search strategy. **Table S1b.** Musculoskeletal disease search strategy. **Table S1c.** Chronic disease search strategy. Additional file 2: Table 2a. Hypertension articles (*n* = 22). **Table S2b.** Ischaemic heart disease articles (*n* = 19). **Table S2c.** Heart failure articles (*n* = 12). **Table S2d.** Lower Back Pain articles (*n* = 6). **Table S2e.** Osteoarthritis articles (*n* = 7). **Table S2f.** Rheumatoid arthritis articles (*n* = 4).

## Abbreviations

CI: Confidence Interval
CVD: Cardiovascular Disease
HF: Heart Failure
IHD: Ischaemic Heart Disease
LBP: Lower Back Pain
MCID: Minimal Clinically Importance Difference
MCS: Mental Component Summary
MeSH: Medical Subject Heading
MI: Myocardial Infarction
MSD: Musculoskeletal Disease
NHS: National Health Service
NIHR: National Institute for Health Research
OA: Osteoarthritis
PCS: Physical Component Summary
RA: Rheumatoid Arthritis
RCT: Randomised Control Trial
SD: Standard Deviation
SE: Standard Error
UK: United Kingdom
US: United States
