# Supplementary material for: Variations in patient-reported physical health between cardiac and musculoskeletal diseases: systematic review and meta-analysis of population-based studies
Source: Health Qual Life Outcomes. 2015 May 30;13:71. doi: 10.1186/s12955-015-0265-x (PMC4448727; doi:10.1186/s12955-015-0265-x)
Supplement: Additional file 1: Table S1a. — Cardiovascular disease search strategy. Table S1b. Musculoskeletal disease search strategy. Table S1c. Chronic disease search strategy. [file 12955_2015_265_MOESM1_ESM.docx]

Supplementary table 1a: Cardiovascular disease search strategy

|  |  | **Hits** | | |
| --- | --- | --- | --- | --- |
| **No.** | **Search** | **MEDLINE** | **EMBASE** | **CINAHL** |
|  |  |  |  |  |
|  | **OUTCOME** |  |  |  |
| 1. | "quality of life*".ti,ab | 103062 | 132545 | 28902 |
| 2. | "health survey*".ti,ab | 13024 | 13934 | 3389 |
| 3. | "short form*".ti,ab | 11938 | 13749 | 3529 |
| 4. | sf-36*.ti,ab | 9223 | 11493 | 2702 |
| 5. | sf36*.ti,ab | 531 | 768 | 2702 |
| 6. | sf-12*.ti,ab | 1396 | 1780 | 435 |
| 7. | sf12*.ti,ab | 123 | 185 | 435 |
| **8.** | **1 OR 2 OR 3 OR 4 OR 5 OR 6 OR 7** | **120042** | **151257** | **33634** |
|  | **SETTING** |  |  |  |
| 9. | exp GENERAL PRACTICE  (*FAMILY PRACTICE/ for Cinahl) | 57526 | 59807 | 4683 |
| 10. | "general population*".ti,ab | 47004 | 56083 | 7006 |
| 11. | “general practice*” | 27433 | 31795 | 4500 |
| 12. | "primary care*".ti,ab | 54762 | 64476 | 22540 |
| 13. | "family practice*".ti,ab | 6867 | 6775 | 1222 |
| 14. | "family medicine*".ti,ab | 5493 | 6028 | 912 |
| **15.** | **9 OR 10 OR 11 OR 12 OR 13 OR 14** | **164441** | **187589** | **36841** |
|  | **POPULATION** |  |  |  |
| 16. | exp CARDIOVASCULAR DISEASES/ | 1614790 | 2274253 | 182295 |
| 17. | cardiovascular*.ti,ab | 207581 | 252284 | 27365 |
| 18. | CVD*.ti,ab | 10695 | 13437 | 2094 |
| 19. | hypertensi*.ti,ab | 265548 | 315187 | 20370 |
| 20. | "high blood pressure*".ti,ab | 8630 | 10417 | 1375 |
| 21. | "heart disease*".ti,ab | 104238 | 120778 | 13117 |
| 22. | angina*.ti,ab | 41356 | 48061 | 2944 |
| 23. | "myocardial infarction*".ti,ab | 111170 | 130901 | 11860 |
| 24. | "heart attack*".ti,ab | 3264 | 3776 | 1457 |
| 25. | "heart failure*".ti,ab | 83119 | 102791 | 12542 |
| **26.** | **16 OR 17 OR 18 OR 19 OR 20 OR 21**  **OR 22 OR 23 OR 24 OR 25** | **1816439** | **2414107** | **201412** |
|  | **COMBINED SEARCHES** |  |  |  |
| **27.** | **8 AND 15 AND 26** | **1042** | **1525** | **276** |
|  | **LIMITED SEARCHES*** |  |  |  |
| **28.** | **27** | **644** | **1215** | **167** |

**Final searches limited by; date (Medline and Embase: 1990-current, Cinahl: 1990-2011); human studies only (available only for Medline & Embase); age (Medline and Cinahl: All adults, Embase: 18-64, 65+) and language (English).*

Supplementary table 1b: Musculoskeletal disorder search strategy

|  |  | **Hits** | | |
| --- | --- | --- | --- | --- |
| **No.** | **Search** | **MEDLINE** | **EMBASE** | **CINAHL** |
|  |  |  |  |  |
|  | **OUTCOME** |  |  |  |
| 1. | "quality of life*".ti,ab | 103062 | 132545 | 28902 |
| 2. | "health survey*".ti,ab | 13024 | 13934 | 3389 |
| 3. | "short form*".ti,ab | 11938 | 13749 | 3529 |
| 4. | sf-36*.ti,ab | 9223 | 11493 | 2702 |
| 5. | sf36*.ti,ab | 531 | 768 | 2702 |
| 6. | sf-12*.ti,ab | 1396 | 1780 | 435 |
| 7. | sf12*.ti,ab | 123 | 185 | 435 |
| **8.** | **1 OR 2 OR 3 OR 4 OR 5 OR 6 OR 7** | **120042** | **151257** | **33634** |
|  | **SETTING** |  |  |  |
| 9. | exp GENERAL PRACTICE  (*FAMILY PRACTICE/ for Cinahl) | 57526 | 59807 | 4683 |
| 10. | "general population*".ti,ab | 47004 | 56083 | 7006 |
| 11. | “general practice*” | 27433 | 31795 | 4500 |
| 12. | "primary care*".ti,ab | 54762 | 64476 | 22540 |
| 13. | "family practice*".ti,ab | 6867 | 6775 | 1222 |
| 14. | "family medicine*".ti,ab | 5493 | 6028 | 912 |
| **15.** | **9 OR 10 OR 11 OR 12 OR 13 OR 14** | **164441** | **187589** | **36841** |
|  | **POPULATION** |  |  |  |
| 16. | exp MUSCULOSKELETAL DISEASES/ | 721821 | 1232186 | 76877 |
| 17. | "musculoskeletal*".ti,ab | 19847 | 23738 | 6969 |
| 18. | MSK*.ti,ab | 758 | 1051 | 68 |
| 19. | arthritis*.ti,ab | 104649 | 120246 | 11496 |
| 20. | disorder*.ti,ab | 558177 | 665965 | 60062 |
| 21. | "neck pain*".ti,ab | 4024 | 4826 | 1674 |
| 22. | "back pain*".ti,ab | 22675 | 28284 | 9008 |
| 23. | osteoarthritis*.ti,ab | 25753 | 31869 | 5255 |
| 24. | “rheumatoid arthritis*”.ti,ab | 65110 | 74627 | 5901 |
| 25. | “joint disease*”.ti,ab | 5943 | 6726 | 443 |
| **26.** | **16 OR 17 OR 18 OR 19 OR 20 OR 21**  **OR 22 OR 23 OR 24 OR 25** | **1284273** | **1857903** | **145249** |
|  | **COMBINED SEARCHES** |  |  |  |
| **27.** | **8 AND 15 AND 26** | **1645** | **2300** | **406** |
|  | **LIMITED SEARCHES*** |  |  |  |
| **28.** | **27** | **1096** | **1886** | **250** |

**Final searches limited by; date (Medline and Embase: 1990-current, Cinahl: 1990-2011); human studies only (available only for Medline & Embase); age (Medline and Cinahl: All adults, Embase: 18-64, 65+) and language (English).*

Supplementary table 1c: Chronic disease search strategy

|  |  | **Hits** | | |
| --- | --- | --- | --- | --- |
| **No.** | **Search** | **MEDLINE** | **EMBASE** | **CINAHL** |
|  |  |  |  |  |
|  | **OUTCOME** |  |  |  |
| 1. | "quality of life*".ti,ab | 103062 | 132545 | 28902 |
| 2. | "health survey*".ti,ab | 13024 | 13934 | 3389 |
| 3. | "short form*".ti,ab | 11938 | 13749 | 3529 |
| 4. | sf-36*.ti,ab | 9223 | 11493 | 2702 |
| 5. | sf36*.ti,ab | 531 | 768 | 2702 |
| 6. | sf-12*.ti,ab | 1396 | 1780 | 435 |
| 7. | sf12*.ti,ab | 123 | 185 | 435 |
| **8.** | **1 OR 2 OR 3 OR 4 OR 5 OR 6 OR 7** | **120042** | **151257** | **33634** |
|  | **SETTING** |  |  |  |
| 9. | exp GENERAL PRACTICE  (*FAMILY PRACTICE/ for Cinahl) | 57526 | 59807 | 4683 |
| 10. | "general population*".ti,ab | 47004 | 56083 | 7006 |
| 11. | “general practice*” | 27433 | 31795 | 4500 |
| 12. | "primary care*".ti,ab | 54762 | 64476 | 22540 |
| 13. | "family practice*".ti,ab | 6867 | 6775 | 1222 |
| 14. | "family medicine*".ti,ab | 5493 | 6028 | 912 |
| **15.** | **9 OR 10 OR 11 OR 12 OR 13 OR 14** | **164441** | **187589** | **36841** |
|  | **POPULATION** |  |  |  |
| 16. | exp CHRONIC DISEASE/ | 195728 | 132276 | 21376 |
| 17. | chronic*.ti,ab | 681346 | 786110 | 64469 |
| **18.** | **16 OR 17** | 735554 | 826533 | 72875 |
|  | **COMBINED SEARCHES** |  |  |  |
| **19.** | **8 AND 15 AND 18** | **1476** | **1788** | **384** |
|  | **LIMITED SEARCHES*** |  |  |  |
| **20.** | **19** | **930** | **1437** | **228** |

**Final searches limited by; date (Medline and Embase: 1990-current, Cinahl: 1990-2011); human studies only (available only for Medline & Embase); age (Medline and Cinahl: All adults, Embase: 18-64, 65+) and language (English)*
