# Supplementary material for: Variations in patient-reported physical health between cardiac and musculoskeletal diseases: systematic review and meta-analysis of population-based studies
Source: Health Qual Life Outcomes. 2015 May 30;13:71. doi: 10.1186/s12955-015-0265-x (PMC4448727; doi:10.1186/s12955-015-0265-x)
Supplement: Additional file 2: Table 2a. — Hypertension articles (n = 22). Table S2b. Ischaemic heart disease articles (n = 19). Table S2c. Heart failure articles (n = 12). Table S2d. Lower Back Pain articles (n = 6). Table S2e. Osteoarthritis articles (n = 7). Table S2f. Rheumatoid arthritis articles (n = 4). [file 12955_2015_265_MOESM2_ESM.docx]

Supplementary Table 1a: Hypertension articles (n = 22)

| **ID** | **Authors** | **Year** | **n** | **Mean age**  **(SD)** | **Country** | **Health Setting** | **Case definition** | **Outcome**  **measure** | **Mean PCS**  **score (SD)** |
| --- | --- | --- | --- | --- | --- | --- | --- | --- | --- |
| 1 | Johnson & Coons* | 1998 | 81 | 52.3 | US | Representative general population sample drawn from White pages and driving licence registrations | Common chronic condition self-report checklist | SF-12 | 44  (10.8) |
| 2 | Ware et al | 1994 | 1689 | 58.4 | US | Representative and stratified general population survey | Chronic condition self-report checklist | SF-12 | 43.68  (10.3) |
| 3 | Erickson et al | 2001 | 125 | 59.0  (11.2) | US | Attendance at either a university general health clinic or a hypertension clinic | Previous diagnosis of hypertension and prescribed anti-hypertensive medication | SF-36 | 49.4  (9.3) |
| 4 | Hobbs et al | 2002 | 1203 | 65.1  (10.0) | UK | 16 general practices from across the West Midlands region of England | Diagnosis in practice records | SF-36 | 47.24 (10.71) |
| 5 | Mena-Martin et al | 2003 | 104 | 59.3  (12.9) | Spain | Random sample of the general population from Valladolid | Self-report of other chronic diseases and physical examination | SF-36 | 46  (11.1) |
| 6 | Alonso et al | 2004 | 665 | 53.5  (17.3) | US | Nationally representative general population | Self-report checklist | SF-36 | 44.57  (11.29) |
| 7 | Alonso et al | 2004 | 448 | 59.4  (15.7) | Italy | Nationally representative general population | Self-report checklist | SF-36 | 44.31  (10.83) |

Supplementary Table 1a: Hypertension articles (n = 22) continued

| 8 | Alonso et al | 2004 | 618 | 60.6  (14.4) | France | Nationally representative general population | Self-report checklist | SF-36 | 43.28  (11.32) |
| --- | --- | --- | --- | --- | --- | --- | --- | --- | --- |
| 9 | Alonso et al | 2004 | 599 | 56.9  (16.2) | Germany | Nationally representative general population | Self-report checklist | SF-36 | 44.19  (11.51) |
| 10 | Alonso et al | 2004 | 308 | 52.6  (15.8) | Norway | Nationally representative general population | Self-report checklist | SF-36 | 44.31  (11.88) |
| 11 | Alonso et al | 2004 | 489 | 54.2  (18.3) | Netherlands | Nationally representative general population | Self-report checklist | SF-36 | 45.81  (10.92) |
| 12 | Alonso et al | 2004 | 316 | 57.1  (16.0) | Denmark | Nationally representative general population | Self-report checklist | SF-36 | 43.91  (12.06) |
| 13 | Alonso et al | 2004 | 502 | 58.7  (13.0) | Japan | Nationally representative general population | Self-report checklist | SF-36 | 44.86  (10.53) |
| 14 | Lam et al* | 2005 | 271 | 42.9 | China | Randomly selected sample of Hong Kong general population | Self-report of Hypertension during telephone survey | SF-36 | 48.06  (9.35) |
| 15 | Schmidt et al* | 2008 | 3410 | 60.5  (11.9) | Germany | Multiple general practices | Clinician selected patients with uncontrolled arterial hypertension | SF-12 | 41.8  (10.4) |
| 16 | Wang et al | 2008 | 217 | 61.5  (13.2) | Germany | Cross-sectional survey across 20 general practices | Record chronic diseases at consultation and self-reported severe illness experience | SF-36 | 42.6  (10.7) |

Supplementary Table 1a: Hypertension articles (n = 22) continued

| 17 | Quercioli et al | 2009 | 218 | 64.8  (12.8) | Italy | General practices in two Italian cities (Turin & Siena) | Self-report of a history of hypertension | SF-36 | 46.4  (8.9) |
| --- | --- | --- | --- | --- | --- | --- | --- | --- | --- |
| 18 | Cunillera et al | 2010 | 917 | 61.1  (18.5) | Spain | Representative general population health survey in the autonomous area of Catalonia | Common chronic condition self-report checklist | SF-12 | 43.2  (14.23) |
| 19 | Grimaldi-Bensouda et al | 2011 | 1111 | 43.3  (22.8) | France | 825 randomly selected GPs from across France were recruited. All patients consulting on a single day were provided a survey | GPs recorded main reason for consultation and up to five conditions. Reasons for consultation were then coded | SF-12 | 43.9  (11.3) |
| 20 | Prior et al | 2011 | 1606 | 68.1  (9.5) | UK | 6 general practices from the north Staffordshire region of England | Diagnosis by clinician, defined by Read code in practice records | SF-12 | 38.38  (12.23) |
| 21 | de la Hoz  Caballer et al | 2012 | 157 | 57.6  (12) | Spain | 74 randomly selected, nationwide primary care centres | Clinician recorded blood pressure greater than systolic 140 mmHG and/or diastolic 95 mmHg on day of visit | SF-36 | 44.7  (10.5) |
| 22 | Zygmuntowicz  et al | 2012 | 12525 | 57  (12) | Poland | 832 primary health care physicians recruited | Patients had treatment for hypertension in the last 3 months | SF-12 | 42.48  (9.56) |

JNC VI: Joint National Committee on Prevention, Detection, Evaluation, and Treatment of High Blood Pressure 7; PCS score: Physical Component Summary score;

SD: Standard Deviation; SF-12: Short-Form 12 Health Survey; SF-36: Short-Form 36 Health Survey; UK: United Kingdom; US: United States of America. *Mean age based on full study sample

Supplementary Table 1b: Ischaemic heart disease articles (n = 19)

| **ID** | **Author, year** | **Year** | **n** | **Mean age**  **(SD)** | **Country** | **Health setting** | **Case definition** | **Outcome**  **measure** | **Mean PCS**  **score (SD)** |
| --- | --- | --- | --- | --- | --- | --- | --- | --- | --- |
| 4 | Hobbs et al | 2002 | 531 | 68.04  (9.58) | UK | 16 general practices from across the West Midlands region of England | Previous diagnosis of angina in practice records | SF-36 | 40.66  (11.09) |
| 4 | Hobbs et al | 2002 | 396 | 67.18  (9.7) | UK | 16 general practices from across the West Midlands region of England | Previous diagnosis of MI in practice records | SF-36 | 43.12  (11.21) |
| 6 | Alonso et al | 2004 | 141 | 60.46  (16.30) | US | Nationally representative general population | Self-reported checklist  of IHD | SF-36 | 37.11  (12.35) |
| 7 | Alonso et al | 2004 | 104 | 66.58  (16.21) | Italy | Nationally representative general population | Self-reported checklist  of IHD | SF-36 | 37.47  (10.17) |
| 8 | Alonso et al | 2004 | 103 | 65.56  (15.18) | France | Nationally representative general population | Self-reported checklist  of IHD | SF-36 | 36.55  (10.76) |
| 9 | Alonso et al | 2004 | 289 | 64.05  (14.32) | Germany | Nationally representative general population | Self-reported checklist  of IHD | SF-36 | 38.33  (11.22) |
| 10 | Alonso et al | 2004 | 47 | 64.06  (11.24) | Norway | Nationally representative general population | Self-reported checklist  of IHD | SF-36 | 40.68  (11.59) |
| 11 | Alonso et al | 2004 | 190 | 55  (20.62) | Netherlands | Nationally representative general population | Self-reported checklist  of IHD | SF-36 | 41.98  (11.98) |
| 12 | Alonso et al | 2004 | 78 | 63.88  (11.75) | Denmark | Nationally representative general population | Self-reported checklist  of IHD | SF-36 | 37.63  (12.83) |

Supplementary Table 1b: Ischaemic heart disease articles (n = 19) continued

| 13 | Alonso et al | 2004 | 100 | 59.26  (14.51) | Japan | Nationally representative general population | Self-reported checklist  of IHD | SF-36 | 41.88  (10.79) |
| --- | --- | --- | --- | --- | --- | --- | --- | --- | --- |
| 23 | Buckley & Murphy | 2009 | 449 | 65.0  (9.09) | Ireland | 37 general practices in the west and north-west of Ireland | IHD diagnosis (excluding angina) in medical records | SF-36 | 42  (8.95) |
| 23 | Buckley & Murphy | 2009 | 275 | 67.2  (9.01) | Ireland | 37 general practices in the west and north-west of Ireland | Angina diagnosis in medical records | SF-36 | 39.38  (9.38) |
| 18 | Cunillera et al | 2010 | 105 | 67.25  (14.96) | Spain | Representative general population health survey in the autonomous area of Catalonia | Self-report of previous MI on common chronic condition checklist | SF-12 | 38.1  (15.06) |
| 19 | Grimaldi-Bensouda et al | 2011 | 96 | 43.3  (22.8) | France | 825 randomly selected GPs from across France were recruited. All patients consulting on a single day were provided a survey | GPs recorded a reason for consultations as other IHD | SF-12 | 41.1  (11.9) |
| 19 | Grimaldi-Bensouda et al | 2011 | 66 | 43.3  (22.8) | France | 825 randomly selected GPs from across France were recruited. All patients consulting on a single day were provided a survey | GPs recorded a reason for consultations as angina | SF-12 | 41.8  (11.8) |

Supplementary Table 1b: Ischaemic heart disease articles (n = 19) continued

| 19 | Grimaldi-Bensouda et al | 2011 | 84 | 43.3  (22.8) | France | 825 randomly selected GPs from across France were recruited. All patients consulting on a single day were provided a survey | GPs recorded a reason for consultations as acute MI | SF-12 | 40.8  (11.4) |
| --- | --- | --- | --- | --- | --- | --- | --- | --- | --- |
| 20 | Prior & Kadam | 2011 | 257 | 69.4  (8.3) | UK | 6 general practices from the north Staffordshire region of England | Defined by IHD Read code in practice records | SF-12 | 33.36  (10.8) |
| 20 | Prior & Kadam | 2011 | 286 | 70.2  (9.5) | UK | 6 general practices from the north Staffordshire region of England | Angina diagnosis by clinician, defined by Read code in practice records | SF-12 | 31.68  (9.78) |
| 20 | Prior & Kadam | 2011 | 44 | 68.9  (9.4) | UK | 6 general practices from the north Staffordshire region of England | Defined by MI Read code in practice records | SF-12 | 34.71  (11.42) |

IHD: Ischaemic Heart Disease; UK: United Kingdom; US: United States of America; SD: Standard Deviation

Supplementary Table 1c: Heart failure articles (n = 12)

| **ID** | **Author, year** | **Year** | **n** | **Mean age**  **(SD)** | **Country** | **Health setting** | **Case definition** | **Outcome**  **measure** | **Mean PCS score (SD)** |
| --- | --- | --- | --- | --- | --- | --- | --- | --- | --- |
| 4 | Hobbs et al | 2002 | 399 | 72.71  (9.49) | UK | 16 general practices from across the West Midlands region of England | Previous diagnosis in practice records | SF-36 | 35.97  (10.13) |
| 24 | Sidorov et al | 2003 | 268 | 75.2 | US | Primary care based, HMO sponsored patient education programme | Health plan members with diagnosis of HF | SF-36 | 33.8  (10.8) |
| 6 | Alonso et al | 2004 | 83 | 64.74  (11.38) | US | Nationally representative general population | Self-report checklist | SF-36 | 31.02  (10.64) |
| 7 | Alonso et al | 2004 | 127 | 66.72  (13.15) | Italy | Nationally representative general population | Self-report checklist | SF-36 | 37.07  (11.09) |
| 8 | Alonso et al | 2004 | 142 | 67.97  (12.62) | France | Nationally representative general population | Self-report checklist | SF-36 | 36.77  (10.86) |
| 9 | Alonso et al | 2004 | 250 | 64.43  (14.30) | Germany | Nationally representative general population | Self-report checklist | SF-36 | 36.63  (11.37) |
| 10 | Alonso et al | 2004 | 91 | 63.52  (12.79) | Norway | Nationally representative general population | Self-report checklist | SF-36 | 37.6  (11.47) |
| 11 | Alonso et al | 2004 | 170 | 62.16  (17.48) | Netherlands | Nationally representative general population | Self-report checklist | SF-36 | 39.6 (12.41) |
| 12 | Alonso et al | 2004 | 35 | 65.26  (12.50) | Denmark | Nationally representative general population | Self-report checklist | SF-36 | 35.07  (12.58) |

Supplementary Table 1c: Heart failure articles (n = 12) continued

| 13 | Alonso et al | 2004 | 50 | 57.40  (14.73) | Japan | Nationally representative general population | Self-report checklist | SF-36 | 42.78  (10.9) |
| --- | --- | --- | --- | --- | --- | --- | --- | --- | --- |
| 25 | Faller et al | 2007 | 231 | 64  (13) | Germany | 2 Wurzburg university medical centres | Diagnosis of HF confirmed by clinical, laboratory or echocardiographic criteria | SF-36 | 36.1  (10.6) |
| 20 | Prior & Kadam | 2011 | 139 | 76.5  (8.2) | UK | 6 general practices from the north Staffordshire region of England | Diagnosis by clinician, defined by Read code in practice records | SF-12 | 28.98  (8.95) |

HF: Heart Failure; US: United States of America; SD: Standard Deviation

Supplementary Table 1d: Lower Back Pain articles (n = 6)

| **ID** | **Author, year** | **Year** | **n** | **Mean age**  **(SD)** | **Country** | **Health setting** | **Case definition** | **Outcome**  **measure** | **Mean PCS score (SD)** |
| --- | --- | --- | --- | --- | --- | --- | --- | --- | --- |
| 26 | Suarez-Almazor  et al | 2000 | 46 | 49.9  (14.8) | Canada | Consecutive patients seen by 2 physicians at outpatient clinics | Consulters for LBP | SF-36 | 29.3  (8.1) |
| 27 | Salaffi et al | 2005 | 127 | 61.3  (13.5) | Italy | 16 general practices from the Marche region of Italy | Defined by pain localized in lower back, either radiating or not | SF-36 | 40.1  (9.8) |
| 28 | Hicks et al | 2008 | 140 | 81.0  (5.5) | US | Population-based survey to residential population in Maryland & North Virginia | Self-report, indicating experience pain on diagram | SF-36 | 43.9  (12.1) |
| 16 | Wang et al | 2008 | 193 | 51.1  (15.2) | Germany | Cross-sectional survey across 20 general practices | Record chronic diseases at consultation and self-reported severe illness experience | SF-36 | 41.4  (10.3) |
| 18 | Cunillera et al | 2010 | 1277 | 50.7  (21.4) | Spain | Representative general population health survey in the autonomous area of Catalonia | Self-report of previous LBP on common chronic condition checklist | SF-12 | 44  (13.58) |
| 20 | Prior & Kadam | 2011 | 650 | 64.4  (10.0) | UK | 6 general practices from the north Staffordshire region of England | Diagnosis by clinician, defined by Read code in practice records | SF-12 | 37.59  (12.11) |

US: United States of America; SD: Standard Deviation

Supplementary Table 1e: Osteoarthritis articles (n = 7)

| **ID** | **Author, year** | **Year** | **n** | **Mean age**  **(SD)** | **Country** | **Health setting** | **Case definition** | **Outcome**  **measure** | **Mean PCS**  **score (SD)** |
| --- | --- | --- | --- | --- | --- | --- | --- | --- | --- |
| 2 | Ware et al | 1994 | 994 | 60.8 | US | Representative and stratified general population survey | Chronic condition self-report checklist | SF-12 | 38.91  (10.13) |
| 27 | Salaffi et al | 2005 | 193 | 61.3  (13.5) | Italy | 16 general practices from the Marche region of Italy | Hand, knee or hip OA defined using ACR criteria | SF-36 | 36.6  (8.2) |
| 29 | Rannou et al | 2007 | 4133 | 67  (10) | France | National sample of general practices by geographical stratification | Consulting for, and radiographic evidence of *knee* or *hip* OA | SF-36 | 31.9  (8.4) |
| 16 | Wang et al | 2008 | 17 | 67.8 | Germany | Cross-sectional survey across 20 general practices | Record chronic diseases at consultation and self-reported severe illness experience | SF-36 | 34.7  (11.1) |
| 30 | Loza et al | 2009 | 1071 | 71 (9) | Spain | Patients consulting general practices in 11 geographic regions | Symptomatic and radiologic *knee* or *hip* OA | SF-12 | 35.1  (8.7) |
| 19 | Grimaldi-Bensouda et al* | 2011 | 324 | 43.3  (21.9) | France | 825 randomly selected GPs from across France were recruited. All patients consulting on a single day were provided a survey | GPs recorded a reason for consultations as OA | SF-12 | 41.1  (10.7) |
| 20 | Prior & Kadam | 2011 | 850 | 68.9  (9.7) | UK | 6 general practices from the north Staffordshire region of England | Diagnosis by clinician,  defined by Read code in  practice records | SF-12 | 33.15  (10.96) |

UK: United Kingdom; SD: Standard Deviation

Supplementary Table 1f: Rheumatoid arthritis articles (n = 4)

| **ID** | **Author, year** | **Year** | **n** | **Mean age**  **(SD)** | **Country** | **Health setting** | **Case definition** | **Outcome**  **measure** | **Mean PCS**  **score (SD)** |
| --- | --- | --- | --- | --- | --- | --- | --- | --- | --- |
| 2 | Ware et al | 1994 | 501 | 57.6 | US | Representative and stratified general population survey | Chronic condition self-report checklist | SF-12 | 40.57  (10.53) |
| 31 | Kahn et al* | 2007 | 490 | 54 (11) | US | Patients consulting across 4 Californian medical centres | At least 3 insurance claims for RA and meeting clinical and laboratory criteria | SF-12 | 37  (9) |
| 32 | Uhlig et al* | 2007 | 936 | 61.3 (14.1) | Norway | Representative sample of RA patients in Oslo from local register | Diagnosis of RA defined by ARA criteria | SF-36 | 35  (12) |
| 33 | Salaffi et al | 2009 | 693 | 53.9 (12.9) | Italy | Outpatient clinic from two Rheumatology departments | Diagnosis of RA defined by ARA criteria | SF-36 | 33.55  (6.43) |

ARA: American Rheumatism Association; RA: Rheumatoid Arthritis; UK: United Kingdom; US: United States of America; SD: Standard Deviation
